# Associations of combined smoking and drinking with subjective and objective olfactory dysfunction: A secondary dataset analysis of NHANES 2013–2014

**DOI:** 10.18332/tid/235830

**Published:** 2026-09-22

**Authors:** Lu He, Jianhong Wang, Yuansheng Rao, Fan Yang

**Affiliations:** 1 Department of Otorhinolaryngology Head and Neck Surgery, Beijing Friendship Hospital, Capital Medical University, Beijing, China; 2 Department of Otorhinolaryngology Head and Neck Surgery, Beijing Anzhen Hospital, Capital Medical University, Beijing, China

**Keywords:** olfactory dysfunction, smoking, alcohol consumption

## Abstract

**INTRODUCTION:**

The association between smoking, alcohol consumption, and olfactory dysfunction (OD) remains inconclusive, and few studies have simultaneously examined their relationships with both subjective and objective OD in large population-based samples. This study aimed to investigate the associations of different smoking and drinking statuses with subjective and objective OD among US adults aged ≥40 years.

**METHODS:**

This secondary dataset analysis of NHANES 2013–2014 included 3376 adults aged ≥40 years who completed the chemosensory questionnaire, smoking and alcohol use questionnaires, and the odor identification test. Subjective OD was based on self-reported olfactory impairments from the NHANES chemosensory questionnaire, and objective OD was assessed using an 8-item odor identification test (score 0–5 indicating impairment). Smoking status was classified based on smoking index, serum cotinine, and time to first cigarette; heavy drinking was defined based on daily drinking habits during any period in life. Multivariable logistic regression models adjusted for demographic factors and OD-related risk factors.

**RESULTS:**

The prevalence of subjective and objective OD was 20.3% and 18.3%, respectively. For subjective OD, significantly elevated adjusted odds were observed for chronic smoker (AOR=1.47; 95% CI: 1.12–1.93), chronic active smoker (AOR=1.55; 95% CI: 1.16–2.08), high dependent active smoker (AOR=1.69; 95% CI: 1.19–2.40), chronic smoker-heavy drinker (AOR=1.72; 95%CI: 1.14–2.60), and active smoker-heavy drinker (AOR=1.90; 95% CI: 1.25–2.88). For objective OD, significantly higher adjusted odds were found for chronic smoker (AOR=1.64; 95% CI: 1.26–2.12), chronic active smoker (AOR=1.59; 95% CI: 1.10–2.32), high dependent smoker-heavy drinker (AOR=2.69; 95% CI: 1.36–5.34), and chronic smoker-heavy drinker (AOR=1.67; 95% CI: 1.12–2.50).

**CONCLUSIONS:**

Chronic smoking and heavy drinking, particularly in combination, are significantly associated with both subjective and objective OD in US adults aged ≥40 years. These findings suggest that smoking and heavy alcohol use may represent modifiable risk factors for olfactory impairment. Further longitudinal studies are warranted to confirm these associations and explore underlying mechanisms.

## INTRODUCTION

The prevalence of olfactory dysfunction (OD) in the general population remains debated, ranging from 3.8% to approximately 50%^1-3^. The causes of OD are manifold, with aging, sinonasal diseases, trauma, and upper respiratory tract infections being the most important; among these, chronic rhinosinusitis is the predominant sinonasal etiology, whereas trauma generally causes OD through injury to the olfactory filae or nasal cavity, and upper respiratory tract infections are the main trigger for postviral olfactory dysfunction^4,5^. On the other hand, OD is associated with neurodegenerative disease such as Alzheimer’s disease and Parkinson’s disease, and has been shown to be an early sign of these diseases^6,7^. Moreover, individuals with OD are more likely to experience environment hazardous exposures, food-related risks and reduced quality of life^8,9^. Cigarette smoking may represent a modifiable risk factor for OD. In animal models, chronic exposure to cigarette smoke decreased functional olfactory receptors^10^. However, findings from population-based studies are less consistent: Murphy et al.^4^ reported current smokers at baseline had higher odds of OD, yet long-term follow-up showed no significant association between baseline smoking status and the incidence of OD^4,11^. Conversely, a German study identified that current smoking as a risk factor for OD, with dose-response relationships observed between cigarettes smoked per day and the frequency of olfactory impairment^12^. Smoking may induce respiratory tract inflammation and structural epithelial changes, including decreased mucociliary activity, goblet cell hyperplasia, and squamous metaplasia, and is thus associated with otitis media, rhinosinusitis, and bronchitis driven by inflammation and impaired ciliary function in the respiratory mucosa^13-15^. Although some studies have pointed to an interaction between smoking and OD, large-sample investigations examining the relationship between smoking and both subjective and objective OD are still lacking, and while smokers are more likely to be heavy drinkers, few studies have focused on their combined effects as potential risk factors for OD^16-18^.

The National Health and Nutrition Examination Survey (NHANES), established and approved by the National Center for Health Statistics (NCHS), is conducted annually to assess the health and nutritional status of the participants in the US population. It employs complex sampling methodology to annually recruit approximately 5000 participants from 15 geographically dispersed clusters^19^. In specific years, NHANES collected data from chemosensory tests and questionnaires, as well as smoking and alcohol use data. Based on the data obtained from NHANES, this study investigated the associations between different levels of smoking and alcohol consumption and both subjective and objective OD.

## METHODS

### Study design

This study utilized demographic data, examination data, and questionnaire data from the 2013–2014 NHANES cycle and conducted a secondary dataset analysis between January and July 2026. The four main component datasets, namely the taste and smell questionnaire data (CSQ), the taste and smell examination data (CSX), the smoking questionnaire (SMQ), and the alcohol use questionnaire (ALQ), were matched and fused based on the unique participant IDs. Among the 10175 participants in the 2013–2014 NHANES cycle, we first excluded 6298 participants aged <40 years and 65 pregnant women. We then excluded participants with missing data on any of the core exposure variables (smoking or alcohol use) or outcome variables (subjective or objective olfactory function). After these exclusions, a final analytic sample of 3376 participants was included.

### Subjective assessment of olfactory dysfunction

The NHANES chemosensory questionnaire included items regarding self-reported olfactory status as well as symptoms, medical treatment and presence of OD-related risk factors. These questions were content-validated and tested to ensure consistency in participant understanding, processing and interpretation. Especially, phantom odor sensation, which is defined as the perception of an odor in the absence of any actual olfactory stimulus, is considered a qualitative form of olfactory dysfunction, alongside quantitative decline in smell ability^20^. Thus, this study used three of these questions to indicate subjective OD: 1) had perceived olfactory problems within the past 12 months (refers to had any problem with ability to smell in the past 12 months regardless of the duration), with response options of ‘yes’, ‘no’, ‘refused’, or ‘don’t know’; 2) had phantom odor sensation (refers to the perception of an unpleasant, bad or burning in the absence of any actual olfactory stimulus), with response options of ‘yes’, ‘no’, ‘refused’, or ‘don’t know’; 3) had changes in olfaction function since the age of 25 years (refers to how would participants rate their current ability to smell compared to when they were 25 years old), with response options of ‘better now’, ‘worse now’, ‘no change’, ‘refused’, or ‘don’t know’^2^. Participants who responded ‘refused’ or ‘don’t know’ to any of these questions (22 in total) were excluded from the subjective OD classification. A positive response – defined as answering ‘yes’ to question 1 or question 3, or answering ‘worse now’ to question 2 – was classified as subjective OD^2^.

### Objective assessment of olfactory dysfunction

The NHANES used a simple but easy-to use eight-item odor identification test to objectively measure olfactory function, which incorporates four nutrient-related odors (chocolate, strawberry, grape, and onion), two warning odors (smoke and natural gas), and two common household odors (leather and soap). Based on the number of correct identifications of odors, the total smell score ranged from 0 to 8. Each correct identification was assigned equal weight (1 point), and the total score was calculated as a simple sum score of 0–5 (≥3 incorrect answers) indicates objective OD, whereas a score of 6–8 is considered normal^2,21^.

### Smoking assessment and classification

The NHANES questionnaire included questions about daily cigarette use, history of cigarette use, length of time being a smoker and time to first cigarette upon waking in the morning (TTFC). For those who have not quit smoking, the years smoked was calculated as the difference between age at enrollment and the initial smoking age; for those who have quit smoking, the years smoked was the difference between the age at quitting and the initial smoking age. Then, the smoking index (cigarette-year, CY) was calculated separately for former and current smokers. For former smokers: CY = years smoked × daily number of cigarettes smoked at the time of quitting. For current smokers: CY = years smoked × average number of cigarettes smoked during the past 30 days. In addition, the serum cotinine was measured in the NHANES mobile examination center. Based on the questionnaire responses and cotinine levels, the smoking status was classified into nine categories (Supplementary file Table 1).

*Smoker, chronic smoker, former smoker and never smoker*

Participants who reported having ever smoked 100 cigarettes in lifetime in the cigarette-use questionnaire were firstly defined as smokers; those with a smoking index ≥200 CY and still smoking were defined as chronic smokers, whereas those with a smoking index ≥200 CY but reported having quit were defined as former smokers^22^. Never smokers were defined as those who reported smoking less than 100 cigarettes in lifetime. Current study combined never smokers and former smokers into a single group (as ‘never/former smoker’) in part of the data analysis.

*Active smoker, chronic active smoker and nonactive smoker*

Participants with serum cotinine ≥10 ng/mL were defined as active smokers, and active smokers with smoking index ≥200 CY were further defined as chronic active smokers. Those with serum cotinine <10 ng/mL were defined as nonactive smokers, and never/former smokers with serum cotinine <10 ng/mL were further defined as never/former-nonactive smokers.

*High dependent smoker and high dependent active smoker*

Participants who reported TTFC <30 minutes were defined as high dependent smokers, and high dependent smokers with serum cotinine ≥10 ng/mL were further defined as high dependent active smokers.

### Alcohol use assessment and classification

The NHANES collected data of participants’ alcohol use reports including current and life-time alcohol use trends. Participants who answered ‘yes’ to there being a time or times in their life when they drank four/five drinks or more on almost every day were defined as heavy drinkers; the remainder were defined as non-heavy drinkers. Combined with smoking status, participants were further classified as chronic smoker-heavy drinker, active smoker-heavy drinker, and high dependent smoker-heavy drinker. Never or former smokers were further classified as never/former smoker and non-heavy drinker, and never/former-nonactive smoker and non-heavy drinker.

### Risk factors of olfactory dysfunction

Potential risk factors of OD were assessed as covariates, including demographic factors, OD-related risk factors and pathologies. The demographic factors included age, gender, race, marital status and education level. Marital status was categorized as married or unmarried. Education level was classified as lower than high school or high school and higher. As for OD-related risk factors and pathologies, self-reported drug use history (yes, no) was extracted from the NAHNES drug use questionnaire. Olfactory-related pathologies included: persistent cold or flu that last for 1-year, persistent dry mouth in past 1-year, tonsils removed, head or face or skull trauma, nasal congestion in past 1-year since allergy or sinusitis and history of ear infection.

### Statistical analysis

Normally distributed continuous variables were described as means with standard deviations (SDs), while non-normally distributed continuous variables were described using median and interquartile range (IQR). Categorical variables were expressed as frequencies and percentages. To examine variations in variable characteristics among groups, we employed t-test and survey Wilcoxon rank-sum test for continuous variables and the chi-squared test for weighted percentages of categorical variables. Univariate associations between both subjective and objective OD and potential risk factors were assessed with survey binary logistic regression. The univariate pairwise comparisons were considered exploratory and hypothesis-generating; therefore, no adjustment for multiple testing was applied. Subsequently, potential risk factors for OD were examined in adjusted logistic regression models in order to explore the multivariate associations between different smoking-drinking status and OD. Covariates were selected based on univariate associations with OD at a threshold of p<0.05, in addition to age and sex, which were included a priori based on their well-established associations with both smoking and olfactory function. For covariates with sporadic missingness, the survey-weighted models applied listwise deletion, using all available data for each specific model. We performed complete-case analyses for the primary models, including only participants with complete data on the exposure, outcome, and covariates in each model. The results of the regression model analyses were presented as odds ratio (OR) or adjusted odds ratio (AOR), along with 95% confidence interval (CI) and corresponding p-values. All analyses accounted for the complex survey design of NHANES by incorporating the primary sampling units, stratification, and MEC examination weights (wtmec2yr). A two-sided p<0.05 was considered statistically significant. All statistical analyses were performed using the R software (version 4.2.2, R Foundation for Statistical Computing, Vienna, Austria) with the survey package, along with MSTATA software (www.mstata.com).

### Ethics

The NHANES cycles were approved by the CDC’s National Center for Health Statistics (NCHS) Research Ethics Review Board (ERB) and all participants provided written informed consent. As this study utilizes publicly available, anonymized NHANES data, it does not require additional ethical review by the Ethics Committee of the authors’ institute.

## RESULTS

In this study, a total of 3376 participants from the NHANES 2013–2014 were included, comprising 1772 females (52.5%) and 1604 males (47.5%). The age distribution was as follows: 918 participants aged 40–49 years (27.2%), 851 aged 50–59 years (25.2%), 842 aged 60–69years (24.9%), 481 aged 70–79 years (14.2%) and 284 aged ≥80 years (8.4%). Detailed characteristics including race, education level, marital status, history of drug use, risk and pathological factors for OD, and distributions of OD are summarized in Table 1. The prevalence of subjective OD was 20.3%, and the prevalence of objective OD was 18.3%. Examination of the agreement between subjective and objective assessments of OD showed that 167 (4.9%) participants were classified as having OD by both measures, 2241 (66.4%) were negative on both, 517 (15.3%) were subjective-positive but objective-negative, and 451 (13.4%) were subjective-negative but objective-positive. The overall agreement was low, with a Cohen’s kappa coefficient of 0.079 (95% CI: 0.042–0.116, p<0.001), indicating a slight agreement beyond chance between subjectively and objectively measured OD.

**Table 1:** Basic characteristics of study participants, a cross-sectional secondary dataset analysis of US adults, NHANES 2013–2014 (N=3376)

*Characteristics*	*n (%)*
**Demographic**	
**Age** (years)	
40–49	918 (27.2)
50–59	851 (25.2)
60–69	842 (24.9)
70–79	481 (14.2)
≥80 years	284 (8.4)
Mean ± SD	59 ± 12
Median (IQR)	59 (49–68)
**Gender**	
Female	1772 (52.5)
Male	1604 (47.5)
**Race**	
Non-Hispanic White	1526 (45.2)
Non-Hispanic Black	675 (20.0)
Non-Hispanic Asian	366 (10.8)
Mexican American	445 (13.2)
Other Hispanic	295 (8.7)
Other race/multiracial	69 (2.0)
**Education level**	
Lower than high school	774 (22.9)
High school and higher	2602 (77.1)
**Marital status**	
Unmarried	1382 (40.9)
Married	1994 (59.1)
**Risk factors and pathologies of olfactory dysfunction**	
**History of drug use**	
No	2429 (71.9)
Yes	947 (28.1)
**Persistent cold/flu last 1-year**	
No	3123 (92.5)
Yes	253 (7.5)
**Persistent dry mouth**	
No	2849 (84.4)
Yes	527 (15.6)
**Tonsils removed**	
No	2504 (74.2)
Yes	872 (25.8)
**Head/face/skull trauma**	
No	2660 (78.8)
Yes	716 (21.2)
**Nasal congestion in past 1-year since allergy or sinusitis**	
No	1853 (54.9)
Yes	1523 (45.1)
**History of ear infection**	
No	2640 (78.2)
Yes	679 (20.1)
Don’t know	57 (1.7)
**Olfactory dysfunction distributions**	
**Subjective olfactory dysfunction**	
No	2692 (79.7)
Yes	684 (20.3)
**Objective olfactory dysfunction**	
No	2758 (81.7)
Yes	618 (18.3)

SD: standard deviation. IQR: interquartile range.

Univariate analysis for subjective OD revealed that race, marital status and drug-use history were the only demographic variables that associated with subjective OD. As for risk factors, those who ever had experienced any of the OD-related pathologies showed associations with subjective OD. As for smoking status, all types of smoking statuses showed associations with subjective OD when compared with their respective control groups. Further analysis combining both smoking and alcohol use status also revealed associations with subjective OD across all comparison pairs (Supplementary file Table 2). As for univariate analysis for objective OD, the demographic pattern for objective OD differed from that observed for subjective OD: objective OD was more likely to be found among older adults, males, and individuals with a level of education lower than high school. Positive history of drug use also showed association with objective OD. Participants who suffered from persistent dry mouth, nasal congestion, or multiple ear infections also exhibited significantly higher odds of objective OD. As for smoking and alcohol use status, associations with objective OD were observed across all comparison groups, except for the comparison pair between high dependent active smokers and never/former-nonactive smokers (Supplementary file Table 3).

**Figure 1:**
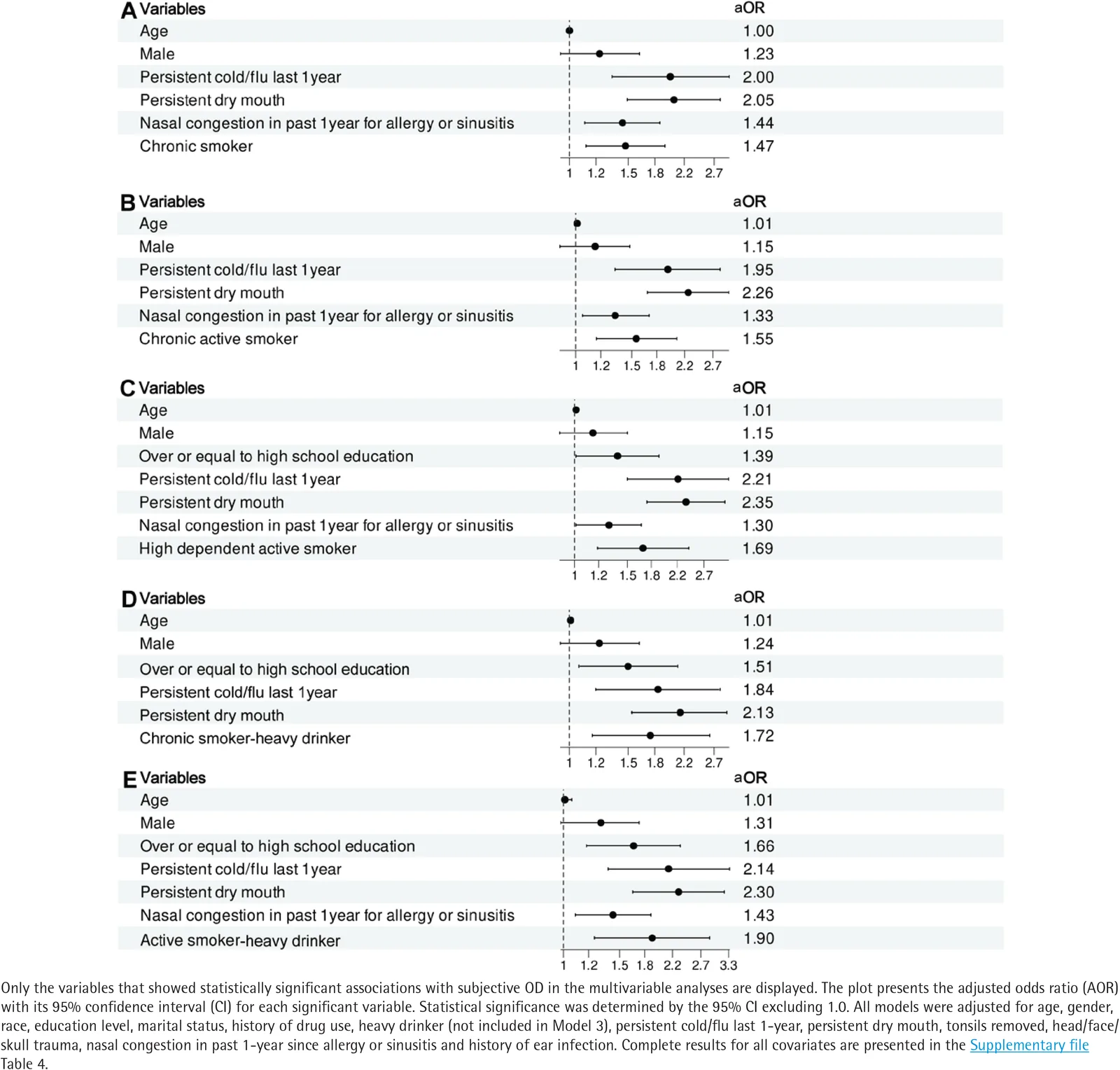
Adjusted odds ratios for subjective olfactory dysfunction by positive smoking–drinking statuses and covariates, NHANES 2013–2014 (N=3376)

**Figure 2:**
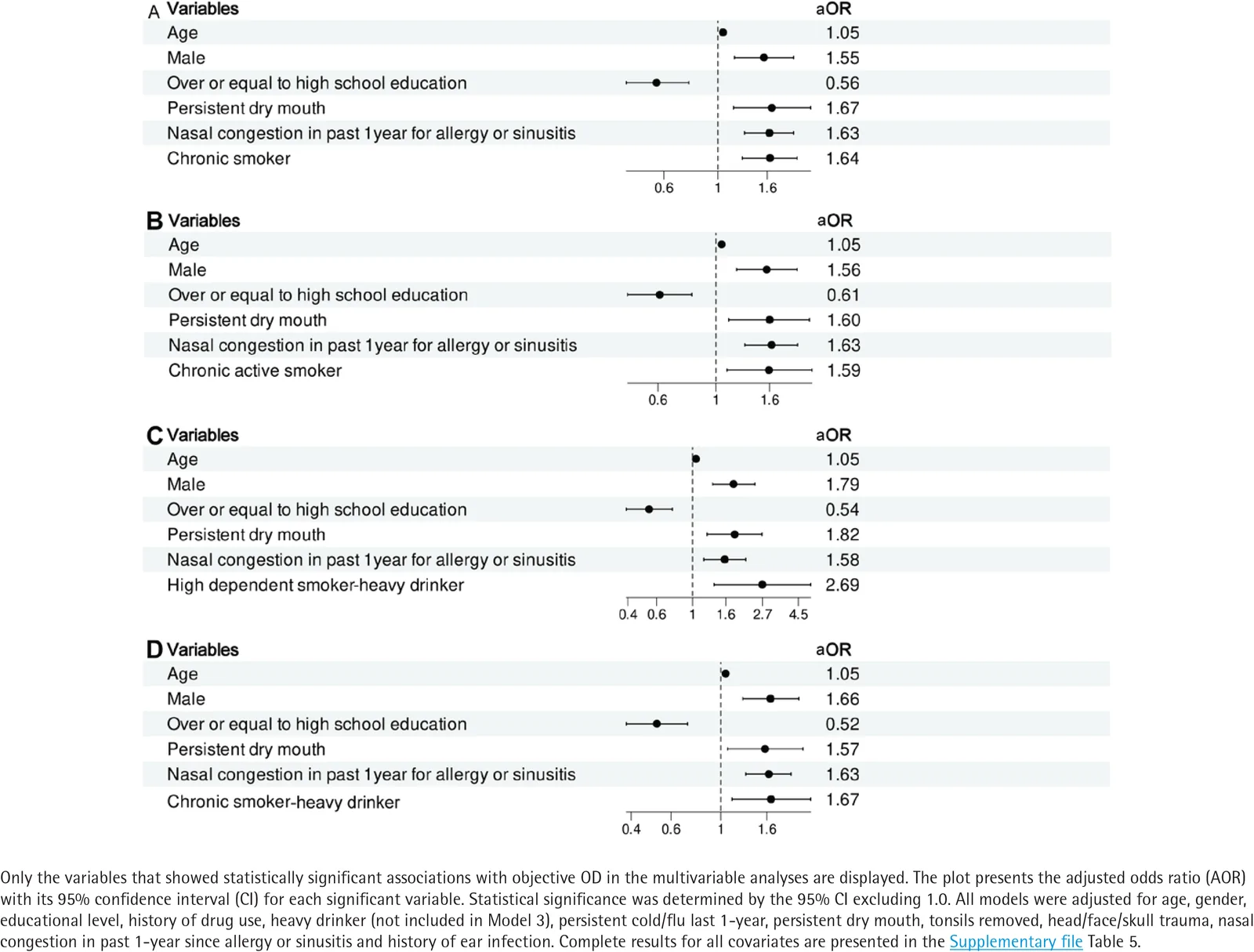
Adjusted odds ratios for objective olfactory dysfunction by positive smoking–drinking statuses and covariates, NHANES 2013–2014 (N=3376)

According to the results of univariate analyses and different smoking-drinking statuses, adjusted multivariate logistic regression models were employed, respectively. To summarize, five of the six smoking-drinking status categories demonstrated consistently elevated adjusted odds ratios for subjective OD, namely: chronic smoker (AOR=1.47; 95% CI: 1.12–1.93), chronic active smoker (AOR=1.55; 95% CI: 1.16–2.08), high dependent active smoker (AOR=1.69; 95% CI: 1.19–2.40), chronic smoker-heavy drinker (AOR=1.72; 95% CI: 1.14-2.60) and active smoker-heavy drinker (AOR=1.90; 95% CI: 1.25–2.88) (Table 2, Figure 1; and Supplementary file Table 4). For objective OD, four smoking–drinking status categories showed significantly higher adjusted odds ratios, namely: chronic smoker (AOR=1.64; 95% CI: 1.26–2.12), chronic active smoker (AOR=1.59; 95% CI: 1.10–2.32), high dependent smoker-heavy drinker (AOR=2.69; 95% CI: 1.36–5.34) and chronic smoker-heavy drinker (AOR=1.67; 95% CI: 1.12–2.50) (Table 3, Figure 2; and Supplementary file Table 5).

**Table 2:** Multivariable adjusted associations between smoking–drinking statuses and subjective olfactory dysfunction, a cross-sectional secondary dataset analysis of US adults, NHANES 2013–2014

*Models and exposure groups^a,b^*	*AOR (95% CI)*	p*
**Model 1: Main group of smoking**		
Chronic smoker (n=2741)	1.47 (1.12–1.93)	0.005
**Model 2: Subgroups of smoking**		
2.1 Chronic active smokers (n=3097)	1.55 (1.16–2.08)	0.003
2.2 High dependent active smokers (n=2926)	1.69 (1.19–2.40)	0.004
**Model 3: Combined groups of smoking and heavy drinking**		
3.1 Chronic smoker-heavy drinker (n=2698)	1.72 (1.14–2.60)	0.010
3.2 Active smoker-heavy drinker (n=2531)	1.90 (1.25–2.88)	0.003
3.3 High dependent smoker-heavy drinker (n=2526)	1.23 (0.97–1.55)	0.088

AOR: adjusted odds ratio. CI: confidence interval.

a Each exposure group was examined in a separate multivariable logistic regression model adjusted for age, gender, race, education level, marital status, history of drug use, heavy drinker (not included in Model 3), persistent cold/flu that last 1-year, persistent dry mouth, tonsils removed, head/face/skull trauma, nasal congestion in past 1-year since allergy or sinusitis and history of ear infection.

b Reference groups for Models 1, 2, 3 (3.1, 3.3) and 3 (3.2) respectively: Never smokers; never/former-nonactive smoker; Never/former smoker and non-heavy drinker and Never/former-nonactive smoker and non-heavy drinker.

* Multivariate analysis results by logistic regression.

**Table 3:** Multivariable-adjusted associations between smoking-drinking statuses and objective olfactory dysfunction, a cross-sectional secondary dataset analysis of US adults, NHANES 2013–2014

*Models and exposure groups^a,b^*	*AOR (95% CI)*	p*
**Model 1: Main group of smoking**		
Chronic smoker (n=2741)	1.64 (1.26–2.12)	0.001
**Model 2: Subgroup of smoking**		
Chronic active smokers (n=3097)	1.59 (1.10–2.32)	0.014
**Model 3: Combined groups of smoking and heavy drinking**		
3.1 High dependent smoker-heavy drinker (n=2526)	2.69 (1.36–5.34)	0.005
3.2 Chronic smoker-heavy drinker (n=2698)	1.67 (1.12–2.50)	0.013
3.3 Active smoker-heavy drinker (n=2531)	1.49 (0.99–2.24)	0.054

AOR: adjusted odds ratio. CI: confidence interval.

a Each exposure group was examined in a separate multivariable logistic regression model adjusted for age, gender, educational level, history of drug use, heavy drinker (not included in Model 3), persistent cold/flu that last 1-year, persistent dry mouth, tonsils removed, head/face/skull trauma, nasal congestion in past 1-year since allergy or sinusitis and history of ear infection.

b Reference groups for Models 1, 2, 3 (3.1 and 3.2) and 3 (3.3) respectively: Never smokers; Never/former-nonactive smoker; Never/former smoker and non-heavy drinker and Never/former-nonactive smoker and non-heavy drinker.

* Multivariate analysis results by logistic regression.

## DISCUSSION

In this study, we analyzed NHANES 2013–2014 data from 3376 US adults aged ≥40 years, to investigate associations between different smoking-drinking statuses and OD. Multivariable-adjusted models demonstrated statistically significant associations between various smoking-drinking statuses and both subjective and objective OD.

Our study revealed an estimated prevalence of subjective OD at 20.3%, which is similar to a previous report^9^. This prevalence may vary mostly by age, since older populations tend to report subjective OD more frequently than middle-aged groups, and this phenomenon may reach a peak of nearly two-thirds among those aged >80 years^4^. Although self-report (i.e. subjective) has been widely accepted as an efficient means of population health surveillance and disease screening, and despite its differences from the objective tests used in NHANES, subjective OD still warrants sufficient attention, particularly given its reported associations with various risk factors and diseases. On the other hand, the prevalence of objective OD was slightly lower than the subjective OD in this study, yet remained somewhat higher than findings from other studies^2,23^. Existing studies have reported that many factors are related to the occurrence of objective OD, such as advanced age, exposure to toxic or harmful substances, head or facial trauma, sinonasal infectious diseases, and upper airway surgeries, which were also observed in this study^14,24,25^. The low agreement between subjective and objective OD observed in our study is consistent with previous findings, suggesting that self-report and objective testing may capture distinct aspects of olfactory function, underscoring the importance of including both assessment modalities in epidemiological research on OD^26^.

Mechanistically, smoking may affect olfactory function by inducing and exacerbating upper airway inflammation. Histological studies of smokers’ olfactory epithelium have found squamous metaplasia, reduced mucociliary activity, mucosa edema and altered morphology of olfactory receptor neurons^27,28^. The reality is that although some reports have suggested that smoking impairs olfactory function through these pathways, few studies have simultaneously examined the associations of both subjective and objective OD with smoking within the same cohort. Existing evidence remains controversial. While most studies^29-31^, such as that by Wälchli et al.^30^ which demonstrated dose- and time-dependent relationships between smoking and objective OD, have reported positive correlations, others have found no association, and some have even suggested a protective effect of smoking on olfactory function. In this study, our main findings confirmed a direct association between smoking and OD, both subjectively and objectively, after controlling for other risk factors. Moreover, we found positive correlations between OD and smoking dependence when incorporating time to first cigarette and cotinine levels into analysis. Time to first cigarette serves as a rapid and inexpensive screening indicator of smoking dependence, and serum cotinine provides additional evidence of smoking dependence. Serum cotinine also played a crucial role in determining whether a participant was in an active smoking period. In baseline comparisons, serum cotinine was higher in participants with subjective OD than in those without, but did not differ by objective OD status. One possible explanation is that a single cotinine measurement captures only recent exposure and is influenced by metabolic variation^32,33^. In multivariable models, however, cotinine-defined smoking categories, for example chronic active smoker, were associated with subjective and objective OD. These findings are not contradictory: the former reflects recent biomarker levels, whereas the latter incorporates cumulative smoking history, which may better capture long-term exposure relevant to olfactory impairment. Currently, a limited number of studies have confirmed that smoking cessation may improve olfactory function whereas passive smoking may damage it^16,17^. Unfortunately, due to insufficient data, we were unable to investigate the relationship between OD and duration of smoking cessation, nor between OD and secondhand smoke exposure.

It is well recognized that smokers are more likely to be heavy drinkers, and that individuals with severe alcohol use disorder frequently exhibit impaired olfaction^18,34^. Alcohol addiction may share similar mechanistic pathways with pathological risk factors for OD and may induce alcohol-related changes in the brain regions important for olfactory processing^11^. To our knowledge, only one study has reported that chronic smokers with heavy alcohol consumption are more likely to report subjective OD^18^. In the present study, we preliminarily analyzed the combined associations of smoking and heavy drinking with olfaction, and found that chronic smokers and/or high-dependent smokers with heavy drinking habits had significantly elevated odds of OD compared with their respective non-smoking and non-heavy-drinking reference groups (as defined in the footnotes of Tables 2 and 3). Besides, we observed that the combination of smoking and heavy drinking showed a stronger trend of both subjective and objective OD compared with smoking alone. However, we have to admit that this study has only revealed the preliminary correlational findings regarding smoking, drinking and OD, and the joint mechanisms underlying their effects on olfaction require further investigation.

### Limitations

This study has several limitations. First, the cross-sectional design precludes causal inferences between smoking, heavy drinking, and OD. Although we adjusted for established confounders, residual confounding from unmeasured variables remains possible. Second, the assessment of subjective OD relied on self-reported measures, which are subject to recall bias and may be influenced by symptom awareness; moreover, the 12-month recall period may have captured transient olfactory episodes rather than persistent dysfunction, potentially introducing outcome misclassification. Third, the low agreement between subjective and objective OD measures suggests that these two assessments capture distinct aspects of olfactory function, which limits direct comparability across outcomes. Fourth, given the exploratory nature of the univariate pairwise comparisons, we did not apply a correction for multiple testing; these findings should be interpreted with caution and considered hypothesis-generating. Fifth, due to limited sample sizes within certain smoking–drinking subgroups, we were unable to examine progressive associations across categories of never smoker, smoker, and smoking-heavy drinker with respect to OD, nor to perform a robust interaction analysis. Sixth, our analysis relied on NHANES-administered questionnaires and assessments, which, while well validated, may lack universal standardization for diagnosing OD or assessing smoking status. Future research should prioritize large-sample longitudinal studies incorporating multimodal objective assessments for both olfactory function and smoking status to better elucidate relationships and their underlying mechanistic pathways.

## CONCLUSIONS

In this secondary dataset analysis of US adults aged ≥40 years, different smoking statuses and heavy drinking, especially when combined, were significantly associated with both subjective and objective OD, suggesting that smoking and heavy alcohol use may be modifiable risk factors for olfactory impairment. These findings support the need for prospective studies to further investigate these associations and explore their potential underlying mechanisms.

## Supplementary Material



## Data Availability

All data utilized in this study can be accessed from the NHANES website at http://www.cdc.gov/nchs/nhanes.
