# Supplementary material for: Associations of combined smoking and drinking with subjective and objective olfactory dysfunction: A secondary dataset analysis of NHANES 2013–2014
Source: Tob Induc Dis. 2026 Sep 22;24:10.18332/tid/235830. doi: 10.18332/tid/235830 (PMC13595438; doi:10.18332/tid/235830)
Supplement: Supplementary file 1 [file TID-24-172-s1.pdf]

Supplementary file

© 2026 He L. et al.

DOI:
10.18332/tid/235830

The content has been provided by the author(s) and has not been reviewed, verified, or endorsed by European Publishing. It may not have undergone peer review. The views, opinions, and recommendations expressed are solely those of the author(s) and do not necessarily reflect the position of European Publishing. European Publishing accepts no responsibility or liability for any consequences arising from the use of, or reliance on, this content.

Supplementary Table 1. Smoking and alcohol use classifications

Characteristic*	N/3376 (%)
**Smoker, chronic smoker, former smoker and never smoker**	
Never smoker	1807/3376 (53.5%)
Former smoker	955/3376 (28.3%)
Chronic smoker	934/3376 (27.7%)
**Active smoker, chronic active smoker and nonactive smoker**	
Nonactive smoker	2617/3376 (77.5%)
Active smoker	759/3376 (22.5%)
Chronic active smoker	480/3376 (14.2%)
**High dependent smoker and high dependent active smoker**	
High dependent smoker	309/3376 (9.2%)
High dependent active smoker	309/3376 (9.2%)
**Alcohol use classification**	

Characteristic*	N/3376 (%)
Non-heavy drinker	2880/3376 (85.3%)
Heavy drinker	496/3376 (14.7%)
**Combined smoking and alcohol use classifications**	
Never/former smoker and non-heavy drinker	2427/3376 (71.9%)
Never/former-nonactive smoker and non-heavy drinker	2326/3376 (68.9%)
Chronic smoker-heavy drinker	271/3376 (8.0%)
Active smoker-heavy drinker	205/3376 (6.1%)
High dependent smoker-heavy drinker	99/3376 (2.6%)

*: The characteristics listed are juxtaposed with each other.

Supplementary Table 2. Distribution and univariate analysis results of subjective OD by different characteristics, NHANES 2013-2014 (n=3376)

**Characteristics**	**Subjective OD**		**Statistics[*]**	***p* value**	**OR (95%CI)**	***p* value[**]**
	**No (n=2692)**	**Yes (n=684)**				
**Age (years, means±SD)**	57.8±11.6	58.2±11.6	0.720	0.483	1.00(1.00-1.01)	0.506
**Age strata (n%)**			1.545	0.216		
40-49 years	729 (29.3%)	189 (26.9%)			Ref.	Ref.
50-59 years	765 (28.6%	176 (32.3%)			1.01(0.80-1.27)	0.961
60-69 years	692 (23.6%)	150 (20.4%)			0.84(0.66-1.06)	0.141
70-79 years	386 (12.4%)	95 (12.7%)			0.95(0.72-1.25)	0.711
≥80 years	210 (6.1%)	74 (7.7%)			1.36(0.99-1.85)	0.052
**Gender (n%)**			0.232	0.637		
Female	1400 (52.4%)	372 (51.4%)			Ref.	Ref.
Male	1292 (47.6%)	312 (48.6%)			0.91(0.77-1.08)	0.266
**Race (n%)**			3.468	0.014		
Other race/multi-racial	46 (1.9%)	23 (2.8%)			Ref.	Ref.
Other Hispanic	352 (6.9%)	93 (7.0%)			0.48(0.27-0.86)	0.013

Mexican American	238 (4.3%)	57 (4.0%)			0.53(0.31-0.93)	0.023
Non-Hispanic white	1,167 (70.8%)	359 (75.5%)			0.62(0.37-1.05)	0.064
Non-Hispanic black	566 (10.8%)	109 (7.9%)			0.39(0.23-0.67)	<0.001
Non-Hispanic Asian	323 (5.3%)	43 (2.7%)			0.27(0.15-0.49)	<0.001
**Educational level (n%)**			0.989	0.336		
Under high school education	625 (15.7%)	149 (13.7%)			Ref.	Ref.
Over or equal to high school education	2,067 (84.3%)	535 (86.3%)			1.09(0.89-1.33)	0.426
**Marital status (n%)**			5.009	0.041		
Currently unmarried	1,074 (34.8%)	308 (38.6%)			Ref.	Ref.
Currently married	1,618 (65.2%)	376 (61.4%)			0.81(0.68-0.96)	0.015
**History of drug use (n%)**			5.357	0.035		
No	1,984 (66.3%)	445 (59.2%)			Ref.	Ref.
Yes	708 (33.7%)	239 (40.8%)			1.51(1.26-1.80)	<0.001
**Persistent cold/flu last one year (n%)**			42.391	<0.001		
No	2,524 (94.4%)	599 (86.5%)			Ref.	Ref.
Yes	168 (5.6%)	85 (13.5%)			2.13(1.61-2.80)	<0.001
**Persistent dry mouth (n%)**			64.390	<0.001		

No	2,359 (89.1%)	490 (75.2%)			Ref.	Ref.
Yes	333 (10.9%)	194 (24.8%)			2.80(2.29-3.43)	<0.001
**Tonsils removed (n%)**			3.530	0.080		
No	2,023 (69.8%)	481 (65.3%)			Ref.	Ref.
Yes	669 (30.2%)	203 (34.7%)			1.28(1.06-1.54)	0.01
**Head/face/skull trauma (n%)**			27.648	<0.001		
No	2,184 (77.1%)	476 (67.9%)			Ref.	Ref.
Yes	508 (22.9%)	208 (32.1%)			1.88(1.55-2.27)	<0.001
**Nasal congestion in past 1 year since allergy or sinusitis (n%)**			13.205	0.002		
No	1,560 (50.5%)	293 (38.0%)			Ref.	Ref.
Yes	1,132 (49.5%)	391 (62.0%)			1.84(1.55-2.18)	<0.001
**History of ear infection (n%)**			5.132	0.014		
No	2,157 (76.5%)	483 (67.1%)			Ref.	Ref.
Yes	494 (21.7%)	185 (31.2%)			1.67(1.37-2.03)	<0.001
Don't know	41 (1.8%)	16 (1.7%)			1.74(0.94-3.07)	0.063
**Heavy drinker (n%)**			4.146	0.060		

No	2,327 (86.0%)	553 (79.7%)			Ref.	Ref.
Yes	365 (14.0%)	131 (20.3%)			1.51(1.21-1.88)	<0.001
**Serum Cotinine [ng/ml, median (IQR)]**	0.02 (0.01, 0.34)	0.03 (0.01, 87.40)	3.723	0.002	1.001(1.001-1.002)	<0.001
**Smoking and drinking status*** (n%)**						
Never smoker	1,505 (68.6%)	302 (53.9%)	22.606	<0.001	Ref.	Ref.
Chronic smoker	692 (31.4%)	242 (46.1%)			1.74(1.44-2.11)	<0.001
Never/former-nonactive smoker	2,114 (85.2%)	503 (78.8%)	28.927	<0.0014	Ref.	Ref.
Chronic active smokers	360 (14.8%)	120 (21.2%)			1.40(1.11-1.76)	0.004
Never/former-nonactive smoker	2,114 (90.6%)	503 (85.1%)	8.609	0.010	Ref.	Ref.
High dependent active smokers	229 (9.4%)	80 (14.9%)			1.47(1.11-1.92)	0.006
Never/former smoker and non-heavy drinker	1,981 (96.6%)	446 (92.6%)	6.539	0.022	Ref.	Ref.
High dependent smoker-heavy drinker	72 (3.4%)	27 (7.4%)			1.67(1.04-2.59)	0.028
Never/former-nonactive smoker and non-heavy drinker	1,899 (92.4%)	427 (86.6%)	8.559	0.010	Ref.	Ref.
Active smoker-heavy drinker	150 (7.6%)	55 (13.4%)			1.63(1.17-2.25)	0.003
Never/former smoker and non-heavy drinker	1,981 (91.0%)	446 (82.2%)	8.920	0.009	Ref.	Ref.

Chronic smoker-heavy drinker	194 (9.0%)	77 (17.8%)		1.76(1.32-2.33)	<0.001

CI: Confidence interval, **IQR**: Interquartile range, **OD**: Olfactory dysfunction, **OR**: Odd ratio, **Ref**: Reference, **SD**: standard deviation

*: t test and survey Wilcoxon rank-sum test for continuous variables, the chi-squared test for weighted percentages of categorical variables.

**: Univariate analysis results by logistic regression,

***: Different smoking and drinking statuses are compared in pairs

Supplementary Table 3. Distribution and univariate analysis results of objective OD by different characteristics, NHANES 2013-2014 (n=3376)

Characteristics	Objective OD		Statistics*	*p* value	OR (95%CI)	*p* value**
	No (n=2758)	Yes (n=618)				
**Age (years, means±SD)**	56.8 ± 11.1	64.2 ± 12.4	14.318	<0.001	1.06(1.05-1.06)	<0.001
**Age strata (n%)**						
40-49 years			48.907	<0.001	Ref.	Ref.
50-59 years	834 (31.0%)	84 (15.1%)			1.55(1.15-2.10)	0.04
60-69 years	736 (30.5%)	115 (22.6%)			2.35(1.77-3.12)	<0.001
70-79 years	681 (22.8%)	161 (23.3%)			3.37(2.49-4.59)	<0.001
≥80 years	359 (11.5%)	122 (18.6%)			9.12(6.62-12.65)	<0.001
**Gender (n%)**			13.979	0.002		
Female	1,520 (53.9%)	252 (41.8%)			Ref.	Ref.
Male	1,238 (46.1%)	366 (58.2%)			1.78(1.49-2.13)	<0.001
**Race (n%)**			2.404	0.074		
Other race/multi-racial	60 (2.3%)	9 (1.3%)			Ref.	Ref.
Other Hispanic	236 (4.1%)	59 (4.9%)			1.67(0.82-3.77)	0.186
Mexican American	369 (6.9%)	76 (7.1%)			1.37(0.68-3.07)	0.403

Non-Hispanic white	1,267 (72.6%)	259 (66.9%)			1.36(0.70-2.98)	0.395
Non-Hispanic black	531 (9.7%)	144 (13.2%)			1.81 (0.92-3.98)	0.109
Non-Hispanic Asian	295 (4.4%)	71 (6.5%)			1.60(0.80-3.60)	0.215
**Educational level (n%)**			33.713	<0.001		
Under high school education	569 (13.6%)	205 (25.3%)			Ref.	Ref.
Over or equal to high school education	2,189 (86.4%)	413 (74.7%)			0.52(0.43-0.63)	<0.001
**Marital status (n%)**			2.389	0.143		
Currently unmarried	1,109 (35.0%)	273 (39.1%)			Ref.	Ref.
Currently married	1,649 (65.0%)	345 (60.9%)			0.85(0.71-1.01)	0.070
**History of drug use (n%)**			38.506	<0.001		
No	1,916 (62.7%)	513 (77.5%)			Ref.	Ref.
Yes	842 (37.3%)	105 (22.5%)			0.47(0.37-0.58)	<0.001
**Persistent cold/flu last one year (n%)**			1.671	0.216		
No	2,564 (92.9%)	559 (91.3%)			Ref.	Ref.
Yes	194 (7.1%)	59 (8.7%)			1.39(1.02-1.88)	0.033
**Persistent dry mouth (n%)**			6.998	0.018		
No	2,345 (87.0%)	504 (80.8%)			Ref.	Ref.

Yes	413 (13.0%)	114 (19.2%)			1.28(1.02-1.61)	0.032
**Tonsils removed (n%)**			1.555	0.231		
No	2,063 (69.4%)	441 (65.4%)			Ref.	Ref.
Yes	695 (30.6%)	177 (34.6%)			1.19 (0.98-1.44)	0.078
**Head/face/skull trauma (n%)**			2.838	0.113		
No	2,189 (75.4%)	471 (72.9%)			Ref.	Ref.
Yes	569 (24.6%)	147 (27.1%)			1.20(0.97-1.47)	0.083
**Nasal congestion in past 1 year since allergy or sinusitis (n%)**			41.586	<0.001		
No	1,425 (44.9%)	428 (65.4%)			Ref.	Ref.
Yes	1,333 (55.1%)	190 (34.6%)			0.47(0.39-0.57)	<0.001
**History of ear infection (n%)**			8.033	0.002		
No	2,136 (73.6%)	504 (79.4%)			Ref.	Ref.
Yes	579 (24.8%)	100 (17.5%)			0.73(0.58-0.92)	0.009
Don't know	43 (1.5%)	14 (3.1%)			1.38(0.72-2.48)	0.302
**Heavy drinker (n%)**			6.468	0.022		
No	2,367 (85.5%)	513 (79.7%)			Ref.	Ref.

Yes	391 (14.5%)	105 (20.3%)			1.24(0.98-1.56)	0.075
**Serum Cotinine [ng/ml, median (IQR)]**	0.02 (0.01, 0.61)	0.03 (0.01, 6.51)	1.808	0.092	1.00(1.000-1.001)	0.040
**Smoking and drinking status[***] (n%)**						
Never smoker	1,506 (67.1%)	301 (55.5%)	8.817	0.010	Ref.	Ref.
Chronic smoker	722 (32.9%)	212 (44.5%)			1.47(1.21-1.79)	<0.001
Never/former-nonactive smoker	2,156 (84.4%)	461 (80.5%)	2.643	0.125	Ref.	Ref.
Chronic active smokers	369 (15.6%)	111 (19.5%)	2.579	0.129	1.41(1.11-1.77)	0.004
Never/former-nonactive smoker	2,156 (89.9%)	461 (86.8%)			Ref.	Ref.
High dependent active smokers	243 (10.1%)	66 (13.2%)			1.27(0.94-1.69)	0.106
Never/former smoker and non-heavy drinker	1,999 (96.3%)	428 (92.8%)	6.783	0.020	Ref.	Ref.
High dependent smoker-heavy drinker	71 (3.7%)	28 (7.2%)			1.84(1.16-2.86)	0.008
Never/former-nonactive smoker and non-heavy drinker	1,923 (91.8%)	403 (87.5%)	2.851	0.112	Ref.	Ref.
Active smoker-heavy drinker	158 (8.2%)	47 (12.5%)			1.42(1.00-1.98)	0.045
Never/former smoker and non-heavy drinker	1,999 (90.0%)	428 (84.3%)	8.338	0.011	Ref.	Ref.
Chronic smoker-heavy drinker	210 (10.0%)	61 (15.7%)			1.36(1.00-1.84)	0.049

CI: Confidence interval, **IQR**: Interquartile range, **OD**: Olfactory dysfunction, **OR**: Odd ratio, **Ref**: Reference, **SD**: standard deviation

*: t test and survey Wilcoxon rank-sum test for continuous variables, the chi-squared test for weighted percentages of categorical variables.

**: Univariate analysis results by logistic regression,

***: Different smoking and drinking statuses are compared in pairs

Supplementary Table 4. Detailed multivariate analysis results of subjective olfactory dysfunction by different characteristics, NHANES 2013-2014 (n=3376)

Characteristics	Model 1[*]		Model 2.1[*]		Model 2.2[*]		Model 3.1[*]		Model 3.2[*]		Model 3.3[*]	
	AOR (95% CI)	*p* value[**]	AOR (95% CI)	*p* value[**]	AOR (95% CI)	*p* value[**]	AOR (95% CI)	*p* value[**]	AOR (95% CI)	*p* value[**]	AOR (95% CI)	*p* value[**]
**Exposure Group**	1.47(1.12-1.93)	0.005	1.55(1.16-2.08)	0.003	1.69(1.19-2.40)	0.004	1.72(1.14-2.60)	0.010	1.90(1.25-2.88)	0.003	1.23(0.97-1.55)	0.088
**Covariates**												
**Age**	1.00(0.99-1.02)	0.486	1.01(0.99-1.02)	0.125	1.01(0.99-1.02)	0.127	1.01(1.00-1.02)	0.204	1.01(0.99-1.02)	0.181	1.01(0.99-1.02)	0.234
**Gender**												
Female	Ref.											
Male	1.23(0.94-1.62)	0.133	1.15(0.89-1.48)	0.277	1.15(0.89-1.50)	0.287	1.24(0.95-1.63)	0.118	1.31(0.98-1.73)	0.064	1.27(0.95-1.69)	0.100
**Race**												
Other race/multi-racial	Ref.											

Other Hispanic	0.90(0.40-2.04)	0.799	0.87(0.40-1.87)	0.715	1.01(0.44-2.28)	0.990	0.88(0.38-2.04)	0.771	1.09(0.45-2.59)	0.853	1.30(0.52-3.27)	0.574
Mexican American	1.02(0.46-2.28)	0.956	0.92(0.43-1.94)	0.823	1.09(0.49-2.41)	0.841	1.04(0.46-2.33)	0.929	1.15(0.50-2.67)	0.744	1.44(0.59-3.55)	0.422
Non-Hispanic white	0.79(0.37-1.67)	0.539	0.72(0.35-1.45)	0.356	0.82(0.38-1.75)	0.607	0.76(0.35-1.65)	0.492	0.81(0.36-1.81)	0.602	1.02(0.43-2.41)	0.973
Non-Hispanic black	0.69(0.32-1.49)	0.340	0.61(0.30-1.27)	0.190	0.68(0.31-1.49)	0.335	0.58(0.26-1.29)	0.184	0.64(0.27-1.44)	0.272	0.73(0.30-1.77)	0.484
Non-Hispanic Asian	0.59(0.26-1.35	0.209	0.53(0.24-1.15)	0.107	0.58(0.25-1.33)	0.195	0.56(0.24-1.30)	0.179	0.59(0.25-1.42)	0.241	0.77(0.31-1.95)	0.585
**Educational level**												
Under high school education						Ref.						
Over or equal to high school education	1.26(0.92-1.71)	0.149	1.32(0.98-1.79)	0.069	1.39(1.01-1.91)	0.042	1.51(1.08-2.12)	0.016	1.66(1.18-2.33)	0.004	1.62(1.14-2.30)	0.007
**Marital status**												

Currently unmarried							Ref.					
Currently married	0.89(0.68-1.17)	0.410	0.98(0.76-1.25)	0.848	0.93(0.72-1.20)	0.583	0.95(0.72-1.25)	0.703	0.84(0.63-1.11)	0.218	0.84(0.63-1.12)	0.229
**History of drug use**												
No							Ref.					
Yes	1.13(0.82-1.56)	0.470	1.26(0.94-1.70)	0.125	1.22(0.90-1.67)	0.199	1.23(0.88-1.72)	0.224	1.31(0.92-1.85)	0.131	1.37(0.97-1.94)	0.072
**Heavy drinker**												
No							Ref.					
Yes	1.25(0.88-1.78)	0.220	1.22(0.87-1.72)	0.251	1.29(0.90-1.85)	0.158	-	-	-	-	-	-
**Persistent cold/flu last one year**												
No							Ref.					

Yes	2.00(1.34-3.00)	<0.001	1.95(1.33-2.85)	<0.001	2.21(1.50-3.26)	<0.001	1.84(1.21-2.81)	0.005	2.14(1.38-3.33)	<0.001	1.92(1.23-2.99)	0.004
**Persistent dry mouth**												
No							Ref.					
Yes	2.05(1.49-2.82)	<0.001	1.22(1.68-3.03)	<0.001	2.35(1.74-3.17)	<0.001	2.13(1.54-2.94)	<0.001	2.30(1.65-3.20)	<0.001	2.04(1.45-2.86)	<0.001
**Tonsils removed**												
No							Ref.					
Yes	1.18(0.89-1.58)	0.251	1.11(0.84-1.45)	0.460	1.13(0.85-1.49)	0.408	1.11(0.83-1.48)	0.500	1.07(0.78-1.45)	0.679	1.09(0.80-1.49)	0.574
**Head/face/skull trauma**												
No							Ref.					
Yes	1.32(0.98-1.76)	0.063	1.27(0.96-1.67)	0.091	1.32(0.99-1.95)	0.059	1.23(0.91-1.67)	0.177	1.22(0.89-1.66)	0.223	1.30(0.95-1.78)	0.103
**Nasal congestion**												

**in past 1 year since allergy or sinusitis**												
No						Ref.						
Yes	1.44(1.11-1.86)	0.006	1.33(1.05-1.70)	0.020	1.30(1.01-1.67)	0.041	1.28(0.96-1.70)	0.090	1.43(1.09-1.88)	0.010	1.53(1.16-2.02)	0.003
**History of ear infection**												
No						Ref.						
Yes	1.21(0.91-1.63)	0.194	1.30(0.99-1.71)	0.063	1.28(0.95-1.71)	0.091	1.23(0.91-1.67)	0.179	1.27(0.93-1.73)	0.132	1.25(0.91-1.72)	0.165
Don't know	0.63(0.29-1.41)	0.263	1.06(0.49-2.28)	0.886	1.11(0.50-2.48)	0.802	1.34(0.61-2.98)	0.465	1.40(0.62-3.15)	0.418	1.44(0.64-3.26)	0.381

AOR: Adjusted odd ratio, **CI**: Confidence interval, **Ref:** Reference

*: Columns represent individual multivariable logistic regression models based on different exposure groups, respectively: Model 1, chronic smoker; Model 2.1, chronic active smoker; Model 2.2, high dependent active smoker; Model 3.1, chronic smoker-heavy drinker; Model 3.2, active smoker-heavy drinker; and Model 3.3, high dependent smoker-heavy drinker. Each exposure group was examined in a separate multivariable

logistic regression model, with the respective reference group as defined in the main text (Table 2 footnotes).

**: Multivariate analysis results by logistic regression

Supplementary Table 5. Detailed multivariate analysis results of objective olfactory dysfunction by different characteristics, NHANES 2013-2014 (n=3376)

Characteristics	Model 1*		Model 2*		Model 3.1*		Model 3.2*		Model 3.3*	
	AOR (95% CI)	*p* value**	AOR (95% CI)	*p* value**	AOR (95% CI)	*p* value**	AOR (95% CI)	*p* value**	AOR (95% CI)	*p* value**
**Exposure Group**	1.64(1.26-2.12)	<0.001	1.59(1.10-2.32)	0.014	2.69(1.36-5.34)	0.005	1.67(1.12-2.50)	0.013	1.49(0.99-2.24)	0.054
**Covariates**										
**Age**	1.05(1.03-1.06)	<0.001	1.05(1.04-1.07)	<0.001	1.05(1.04-1.07)	<0.001	1.05(1.04-1.07)	<0.001	1.05(1.04-1.07)	<0.001
**Gender**										
Female					Ref.					
Male	1.55(1.17-2.05)	0.002	1.56(1.20-2.04)	0.001	1.79(1.33-2.42)	<0.001	1.66(1.25-2.22)	<0.001	1.93(1.43-2.60)	<0.001
**Educational level**										
Under high school education					Ref.					

Over or equal to high school education	0.56(0.42-0.76)	<0.001	0.61(0.46-0.81)	<0.001	0.54(0.39-0.75)	<0.001	0.52(0.38-0.71)	<0.001	0.48(0.35-0.96)	<0.001
**History of drug use**										
No					Ref.					
Yes	0.81(0.53-1.23)	0.315	0.79(0.53-1.18)	0.243	0.67(0.41-1.12)	0.126	0.72(0.45-1.14)	0.161	0.58(0.35-0.96)	0.033
**Heavy drinker**										
No					Ref.					
Yes	1.12(0.76-1.67)	0.563	1.18(0.82-1.69)	0.373	-	-	-	-	-	-
**Persistent cold/flu last one year**										
No					Ref.					
Yes	1.22(0.73-	0.444	1.18(0.72-	0.508	1.13(0.64-	0.670	1.11(0.64-	0.713	1.22(0.68-	0.514

	2.02)		1.93)		2.00)		1.93)		2.19)	
**Persistent dry mouth**										
No					Ref.					
Yes	1.67(1.16-2.41)	0.006	1.60(1.12-2.27)	0.009	1.82(1.23-2.68)	0.003	1.57(1.07-2.31)	0.021	1.75(1.18-2.60)	0.006
**Tonsils removed**										
No					Ref.					
Yes	1.10(0.81-1.49)	0.561	1.04(0.78-1.38)	0.805	1.14(0.82-1.57)	0.434	1.12(0.82-1.53)	0.477	1.11(0.80-1.55)	0.524
**Head/face/skull trauma**										
No					Ref.					
Yes	1.30(0.94-1.80)	0.115	1.25(0.92-1.71)	0.159	1.15(0.79-1.66)	0.474	1.20(0.85-1.70)	0.292	1.03(0.71-1.50)	0.875
**Nasal congestion in past 1 year**										

**since allergy or sinusitis**										
No					Ref.					
Yes	1.63(1.29-2.05)	<0.001	1.63(1.29-2.05)	<0.001	1.58(1.17-2.13)	0.003	1.63(1.29-2.05)	<0.001	1.63(1.29-2.05)	<0.001
**History of ear infection**										
No					Ref.					
Yes	0.93(0.65-1.32)	0.674	0.86(0.61-1.21)	0.387	0.71(0.47-1.06)	0.092	0.76(0.52-1.12)	0.162	0.75(0.50-1.12)	0.160
Don't know	1.94(0.89-4.24)	0.098	1.96(0.97-3.99)	0.063	1.99(0.93-4.26)	0.075	2.20(1.02-4.71)	0.044	2.13(1.01-4.50)	0.047

AOR: Adjusted odd ratio, **CI**: Confidence interval, **Ref:** Reference

*: Columns represent individual multivariable logistic regression models based on different exposure groups, respectively: Model 1, chronic smoker; Model 2, chronic active smoker; Model 3.1, high dependent smoker-heavy drinker; Model 3.2, chronic smoker-heavy drinker; and Model 3.3, active smoker-heavy drinker Each exposure group was examined in a separate multivariable logistic regression model, with the respective reference group as defined in the main text (Table 3 footnotes).

**: Multivariate analysis results by logistic regression
